# Transcriptomics and metabolomics analyses provide insights into resistance genes of tree ferns

**DOI:** 10.3389/fgene.2024.1398534

**Published:** 2024-06-10

**Authors:** Weicheng Yang, Qinqin He, Lijun Zhang, Jiaxing Xiao, Jiao Yang, Bingjie Che, BingChen Zhang, Handan Chen, Jiang Li, Yu Jiang

**Affiliations:** ^1^ School of Life Sciences, Guizhou Normal University/Institute of Karst Caves, Guizhou Normal University, Guiyang, China; ^2^ Guizhou Chishui Alsophila National Nature Reserve Administration, Chishui, China; ^3^ Science and Technology Branch, Guizhou Normal University, Guiyang, China; ^4^ Biozeron Shenzhen, Inc., Shenzhen, China

**Keywords:** metabolomics, transcriptomics, tree ferns, resistance genes, differential metabolites, differential expression

## Abstract

As ancient organisms, tree ferns play a crucial role as an evolutionary bridge between lower and higher plant species, providing various utilitarian benefits. However, they face challenges such as overexploitation, climate change, adverse environmental conditions, and insect pests, resulting in conservation concerns. In this study, we provide an overview of metabolic and transcriptomic resources of leaves in two typical tree ferns, *A. spinulosa* and *A. metteniana*, and explore the resistance genes for the first time. The landscape of metabolome showed that the compound skimmin may hold medicinal significance. A total of 111 differentially accumulated metabolites (DAMs) were detected, with pathway enrichment analysis highlighting 14 significantly enriched pathways, including 2-oxocarboxylic acid metabolism possibly associated with environmental adaptations. A total of 14,639 differentially expressed genes (DEGs) were found, among which 606 were resistance (R) genes. We identified *BAM1* as a significantly differentially expressed R gene, which is one of the core genes within the R gene interaction network. Both the maximum-likelihood phylogenetic tree and the PPI network revealed a close relationship between *BAM1*, *FLS2*, and *TMK*. Moreover, *BAM1* showed a significant positive correlation with neochlorogenic acid and kaempferol-7-*O*-glucoside. These metabolites, known for their antioxidant and anti-inflammatory properties, likely play a crucial role in the defense response of tree ferns. This research provides valuable insights into the metabolic and transcriptomic differences between *A. spinulosa* and *A. metteniana*, enhancing our understanding of resistance genes in tree ferns.

## Introduction

Tree ferns (Cyatheaceae) have attracted significant attention due to their diverse utility values, mainly distributed in mainland China, Southeast Asia and southern Japan. These ancient organisms, predating dinosaurs, have existed on Earth for hundreds of millions of years, preceding the evolution of flowering or cone-bearing plants (Cleal and Thomas, 2009). During the Carboniferous period 300–360 million years ago, tree ferns were a prominent component of the Earth’s flora when conditions for plant growth were optimal. Being the closest lineage to seed plants, tree ferns represent an ancient and highly diverse group (Pryer et al., 2001). Throughout their extensive evolutionary history, tree ferns have adapted to changes in paleogeographic environments, developing mechanisms to cope with environmental stresses, thereby contributing to further adaptive evolution. In addition to their evolutionary significance, tree ferns exhibit high ornamental values and serve as a source of natural products with pharmaceutical applications (Cao et al., 2017). Traditional medicine utilizes tree ferns to treat various health conditions, including bacterial skin infections, kidney diseases, hemorrhoids, varicose veins, and diabetes (Chaparro-Hernández et al., 2022). Metabolites in *Alsophila spinulosa* have been identified for their anti-tumor and antibacterial properties (Ying et al., 2011; Longtine and Tejedor, 2018).

*A. spinulosa* represents a typical species of tree fern. Studies on *A. spinulosa*, including genome *de novo* assembly (Huang et al., 2022), complete chloroplast genome sequencing (Gao et al., 2009), and full-length transcriptomes (Hong et al., 2022), provide essential groundwork for further investigations. Notably, the main differences between *A. spinulosa* and *A. metteniana* Hance are evident in various aspects such as plant morphology, leaves, petioles, branches, and distribution areas. *A. spinulosa* typically grows to a stem height of 6 m or higher, with a diameter ranging from 10 to 20 cm, displaying a tree-like structure. Its leaves are thin and deeply lobed, while the branches are brown and thorny, commonly inhabiting ravines, mountain forests close to water sources, and secluded areas. In contrast, *A. metteniana* plants are generally shorter, around 2 m in height, featuring thicker leaves with a distinct waxy layer and shallow lobes. The branches of *A. metteniana* are darker, and these plants are typically found in relatively damp dense forests or crevices among rocks. The endangerment of *A*. *spinulosa* and *A*. *metteniana* Hance by plant-eating insects poses a significant threat to their growth. Insect mouthpart density varies between the two plant species, influenced by factors like host defense responses and insect oviposition selection. Land plant genomes harbor a class of genes collectively known as Resistance (R) genes, comprising tens to hundreds of genes per genome. R genes are essential for plant defense against various biotic stresses, insect pests, and pathogens (Kaloshian, 2004). To our knowledge, the R genes in *Alsophila* species have not yet been reported.

High-throughput omics techniques, specifically transcriptomics and metabolomics, have become increasingly prevalent in scientific research (Raza, 2020). By predicting and integrating metabolite-protein interactions, a deeper understanding of central regulatory mechanisms can be achieved (Yang et al., 2023). Integrating metabolome and transcriptome data has been explored in multiple plants, including tomato, ginkgo, and peanut (Bai et al., 2021; Lu et al., 2021; Li et al., 2022a). Gene networks play a crucial role in various organisms and systems, effectively revealing the fundamental principles of numerous biological processes and reactions within organisms (Zhao et al., 2021a). In this study, we present an analysis of transcriptomics and metabolomics data obtained from leaf tissues of the tree ferns *A. spinulosa* and *A. metteniana*. Our investigation aims to characterize differentially expressed genes and metabolites, as well as explore the interactions and evolutionary relationships of R genes. This study provides insights into the plant resistance mechanisms of tree ferns.

## Materials and methods

### Experimental materials and tissue collection

The fresh leaves of *A. spinulosa* and *A. metteniana* were gathered in the afternoon of 20 December 2020, at Chishui alsophila national nature Reserve (109°45'∼ 106° 03′n, 28°23'∼ 28° 27′e), Chishui, Guizhou Province, P. R. China. The samples were placed in microcentrifuge tubes, rapidly frozen in liquid nitrogen, and stored at −80°C until metabolite and RNA isolation. Three biological replicates were included for each plant.

### RNA extraction, library preparation, and sequencing

Total RNA was extracted using TRIzol reagent kit (Invitrogen, Carlsbad, CA, USA) according to the manufacturer’s protocol. Degradation and contamination of RNA were verified using 1% RNase-free agarose gel electrophoresis, and the purity and integrity of RNA was assessed on an Agilent 2,100 Bioanalyzer (Agilent Technologies, Palo Alto, CA, USA). High-quality RNA (RNA Integrity Number [RIN] scores >7.5) was used for subsequent experiments. The messenger RNA (mRNA) was enriched using Oligo(dT) beads, followed by fragmentation into short fragments using fragmentation buffer and reverse transcribed into cDNA with random primers. Second-strand cDNA was synthesized by DNA polymerase I, RNase H, dNTP and buffer. Subsequently, the cDNA fragments were purified with QiaQuick PCR extraction kit (Qiagen, Venlo, Netherlands), end repaired, A base added, and ligated to Illumina sequencing adapters. The ligation products were size selected (∼300 bp) by agarose gel electrophoresis, PCR amplified, and sequenced using Illumina NovaSeq 6,000 by Gene Denovo Biotechnology Co. (Guangzhou, China).

### Quantification of gene expression level and differential expression analysis

RNAseq raw reads were trimmed by fastp (v 0.18.0) (Chen et al., 2018) to remove adapter contamination and reads with high uncertainty (N > 10%) or low base quality with default parameters. The gene expression levels were quantified and differential expression analysis was conducted based on the reference genome of *A*. *spinulosa* (Huang et al., 2022), following methods described in previous studies (Sun et al., 2020). Briefly, index of the reference genome was built and paired-end clean reads were aligned to the reference genome using Hisat2 (v 2.2.1) (Kim et al., 2015) with parameters of “--sensitive --no-discordant --no-mixed -I 1 -X 1000”. The reads numbers matrix was generated using htseq (v 0.12.4) (Anders et al., 2015). We used fragments per kilobase of exon model per million reads mapped (FPKM) algorithm (Roberts et al., 2011) to obtain transcriptional profile. Differentially expressed genes (DEGs) of *A. metteniana* (H) vs. *A. spinulosa* (S) was identified using the DEseq2 R package (Love et al., 2014). Genes with FDR values ≤0.05 and FPKM values showing at least a 2-fold difference among samples were considered as DEGs. The hierarchical cluster was performed using R software with hclust function.

### GO and KEGG enrichment analysis of differentially expressed genes

The Gene Ontology (GO) enrichment analysis and Kyoto Encyclopedia of Genes and Genomes (KEGG) pathway analysis were using clusterProfiler (Wu et al., 2021).

### Identification and analysis of differential metabolites

The Q1, Q3, retention time, declustering potential (DP) and collision energy (CE) were used for metabolite identification. The SCIEX OSV1.4 software was used to open the downtime mass spectrum file, and the chromatographic peaks were integrated and corrected. The chromatographic peaks were screened according to the minimum peak height of 500, signal-to-noise ratio of 5, smoothing number of 1 and other information. The peak area of each chromatographic peak represents the relative content of the corresponding substance. Finally, the integral data of all chromatographic peaks are derived to obtain the qualitative and quantitative results of metabolites (Bai et al., 2021). These metabolites were annotated using the KEGG database (Kanehisa and Goto, 2000), HMDB database (Wishart et al., 2013), and Lipidmaps database (Zhu et al., 2013), separately. PLS-DA analysis was applied to calculate the corresponding variable importance in projection (VIP) value. The condition of differential metabolites was VIP value of the PLS-DA model ≥1 and independent sample *t*-test’s *p*-value ≤0.05. Heatmap clustering analysis was performed in the R software with ComplexHeatmap package. KEGG pathway enrichment analysis of DAMs was examined using KOBAS (v2.0.12) (Xie et al., 2011). DAMs were considered to be significantly enriched in metabolic pathways when their *p*-values were <0.05.

### Correlation between genes and metabolites

We adopted a similar method as described in Yang, Sun et al. (Yang et al., 2023) to integrate metabolomics and transcriptomic analyses. The expression correlation between genes and metabolites was evaluated using ‘cor.test’ function under R software (v 3.5.1). We deemed a correlation significant if the absolute value of Pearson correlation coefficient ≥0.9 with a corresponding *p*-value ≤0.01.

### The identification, protein interaction network, and phylogenetic tree of R genes

As genomic data for *A. metteniana* is not available, we used the gene set of *A. spinulosa* as a reference to study R genes. Using a method similar to that described in the eggplant genome study (Li et al., 2021), we employed the RGAugury pipeline (Li et al., 2016) to screen the entire gene set for R gene prediction. The default *p*-value cutoff for initial R gene filtering was set to le-5 for BLASTP. Protein-protein interaction (PPI) network was analyzed using the Search Tool for the Retrieval of Interacting Gene (STRING) database (Szklarczyk et al., 2018), which included direct and indirect associations of proteins. The hub gene was determined using cytoHubba (Chin et al., 2014) with the MCC algorithm. The amino acid sequences of R genes were computed multiple sequence alignments using MAFFT (v 7.505) (Nakamura et al., 2018). Subsequently, the maximum-likelihood phylogenetic tree was obtained by using iqtree (v 2.0.6) (Minh et al., 2020) with parameters of ‘-bb 1,000 -pre iqtree -nt AUTO -m MFP -bnni’. The best model inferred by iqtree was “VT + R9”. The tree was displayed using Interactive Tree Of Life (Letunić and Bork, 2021).

### Validation of gene expression using quantitative real-time PCR

Quantitative real-time PCR (qRT-PCR) has been emerged as an effective method to verify gene expression. We performed qRT-PCR experiment using a similar approach in the yellowhorn transcriptome (Zhao et al., 2021b). Total RNAs were reverse-transcribed using the PrimeScript first-strand cDNA synthesis kit (Takara, Dalian, China). Specific primers were designed using Primer-Blast tools (Ye et al., 2012). The qRT-PCR experiment was carried out using SYBRGreen Fast qPCR Master Mix (Sangon Biotech, China) on an ABI StepOne Plus Real-Time System (ABI, USA), following the manufacturer’s instructions. The quantitative PCR reaction conditions were as follows: 95°C for 90 s, followed by 95°C for 5 s, 60°C for 15 s, and 72°C for 20 s (45 cycles). Three biological replicates were included for each gene. *Actin* was used as the internal reference gene to normalize the qRT-PCR expression data. The 2^−ΔΔCT^ method (Livak and Schmittgen, 2001) was employed to calculate the relative mRNA abundance in each sample for every gene. Finally, we estimated the Pearson correlation coefficient of gene expression between the qRT-PCR and RNA-seq profiles in R software with “cor” function.

## Results

### Metabolome profiling of *A. spinulosa* and *A. metteniana*

**FIGURE 1:**
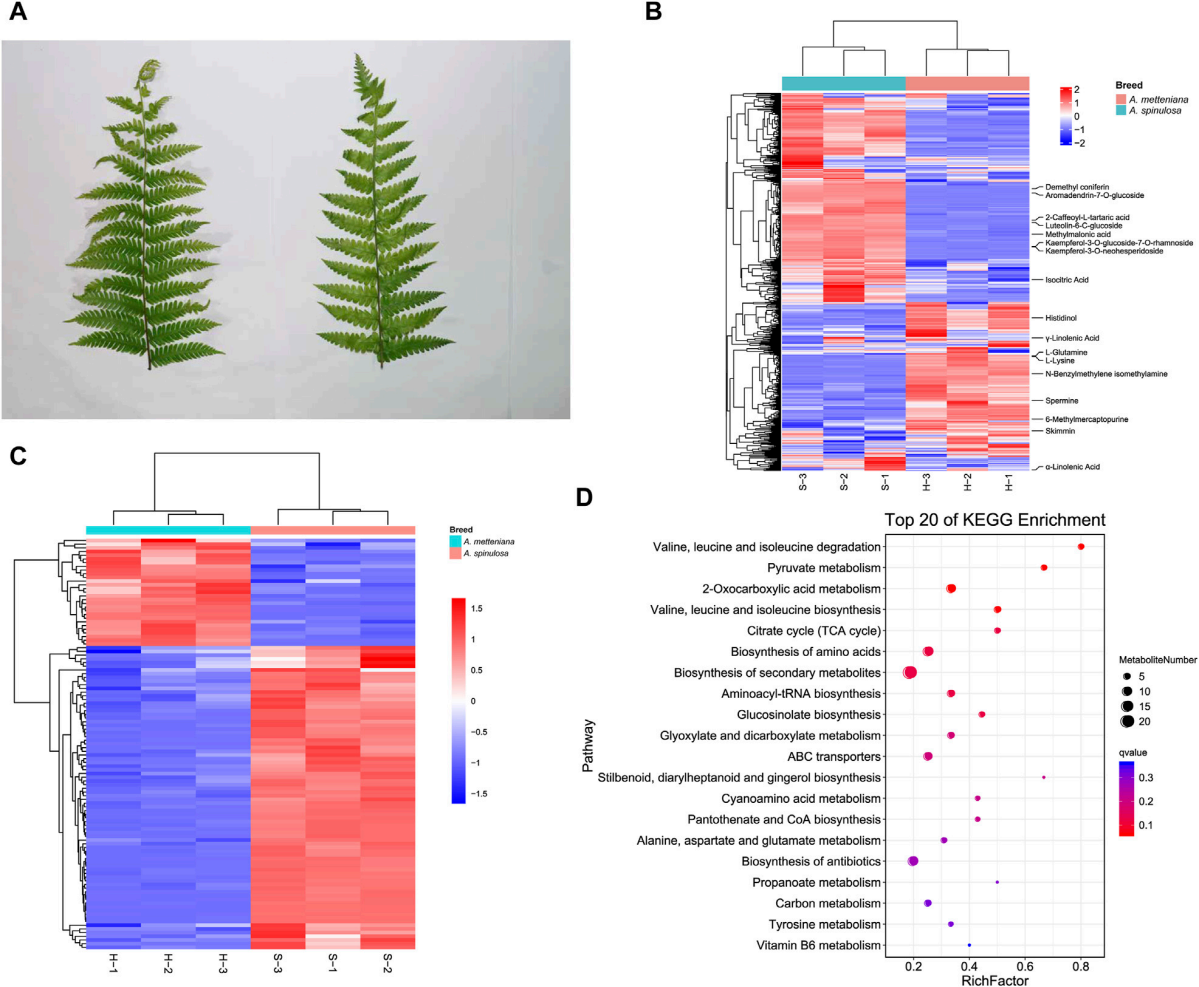
Metabolome profiling of *A. spinulosa* (S) and *A. metteniana* (H). **(A)** The leaves of *A. spinulosa* and *A. metteniana*. **(B)** The heatmap of metabolites, each column represents a sample, and each row represents a metabolite. **(C)** The heatmap of DAMs, each column represents a sample, and each row represents a DAM. The DAM clustering tree is shown on the left. The color scale shown on the right illustrates the relative expression level across all samples: red color represents high expression level, blue color represents low expression level. **(D)** Scatter plot for top 20 KEGG enrichment pathways of DAMs. The *X*-axis label represents rich factor. The rich factor is the ratio of DAMs numbers annotated in this pathway term to all metabolite numbers annotated in this pathway term. The greater the rich factor, the greater the degree of pathway enrichment. A qvalue is the corrected *p*-value ranging from 0 to 1, and a lower value indicates greater pathway enrichment.

After collecting fresh leaves from *A. spinulosa* (S) and *A. metteniana* (H) (Figure 1A), the metabolic components were detected and analyzed. To improve the accuracy of analysis, each group included three replicates. We identified 373 and 399 metabolites in positive and negative modes, respectively (Supplementary Table S1). In *A. spinulosa*, the top 10 metabolites with highest metabolite abundance were “2-Caffeoyl-L-tartaric acid ", “Isocitric Acid”, “Kaempferol-3-*O*-glucoside-7-*O*-rhamnoside”, “Kaempferol-3-*O*-neohesperidoside”, “Skimmin ", “Luteolin-6-C-glucoside ", “Demethyl coniferin”, "γ-Linolenic Acid”, “Methylmalonic acid”, and “Aromadendrin-7-*O*-glucoside”. In *A. metteniana*, the top 10 metabolites with highest metabolite abundance were “Skimmin”, “Isocitric Acid”, “6-Methylmercaptopurine”, "γ-Linolenic Acid”, "α-Linolenic Acid”, “Spermine”, “Histidinol”, “N-Benzylmethylene isomethylamine”, “L-Glutamine” and “L-Lysine” (Figure 1B). The quantity of DAMs was statistically analyzed (Supplementary Table S2). Clustering heatmaps of DAMs showed significant differences in 111 metabolites. Of these, 82 DAMs were upregulated, and 29 metabolites were downregulated (Figure 1C). To verify the function of the DAMs, KEGG pathway enrichment was conducted. A total of 14 pathways were significantly enriched, including “valine, leucine and isoleucine degradation”, “pyruvate metabolism”, “2-Oxocarboxylic acid metabolism”, “valine, leucine and isoleucine biosynthesis”, “citrate cycle (TCA cycle)”, “biosynthesis of amino acids”, “biosynthesis of secondary metabolites”, “aminoacyl-tRNA biosynthesis”, “glucosinolate biosynthesis”, “glyoxylate and dicarboxylate metabolism”, “ABC transporters”, and so on (Figure 1D). Notably, the pathway with the most DAM enrichment pathways was the biosynthesis of secondary metabolites, which had 21 DAMs.

### Transcriptomics analysis of *A. spinulosa* and *A. metteniana*

**FIGURE 2:**
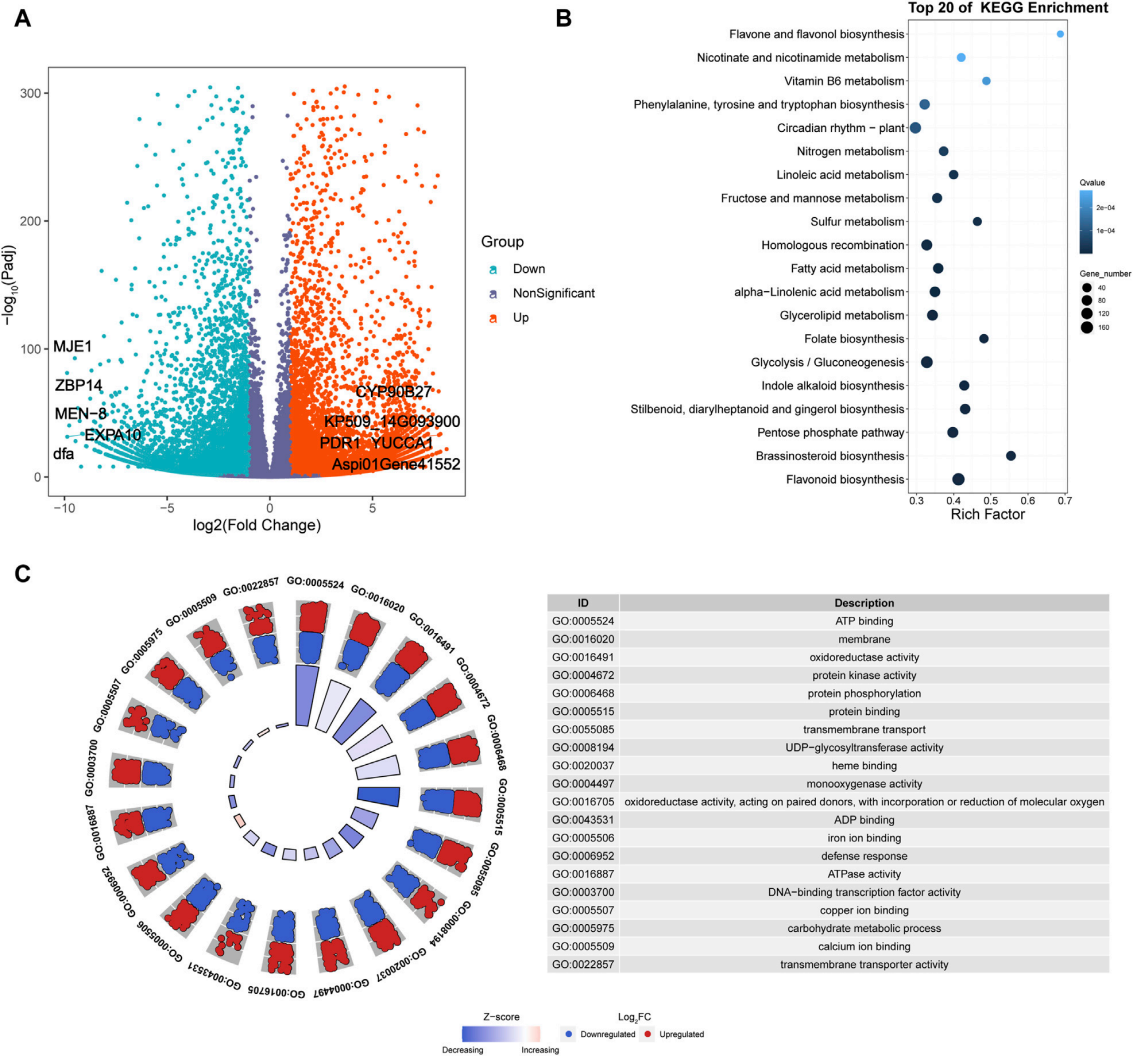
Transcriptomics analysis of *A. spinulosa* and *A. metteniana*. **(A)** Volcano plots illustrate the magnitude and significance of DEGs. X-and *Y*-axis present the log 2 (Fold Change) for the two groups and -log10(padj), respectively. Red (Upregulated) and blue (Downregulated) dots mean that the genes have significant difference, while the dark blue-gray dots correspond to genes with no significant differences. **(B)** Scatter plot for top 20 KEGG enrichment pathways of DEGs. The *X*-axis label represents rich factor. The rich factor is the ratio of differentially expressed gene numbers annotated in this pathway term to all gene numbers annotated in this pathway term. The greater the rich factor, the greater the degree of pathway enrichment. The *Y*-axis label represents pathway. The size and color of the bubble represent the amount of DEGs enriched in the pathway and the enrichment significance, respectively. A Q value is the corrected *p*-value ranging from 0 to 1, and a lower value indicates greater pathway enrichment. **(C)** GO enrichment circle plots for the top 20 most significant GO categories. The outer circle shows the relative fold change for each significant RNA feature compared to air contributing to the GO term with blue dots showing downregulated RNA features and red dots showing upregulated features. The z-score color scale represents the number of upregulated genes minus the number of downregulated genes for a given GO term divided by the square root of the total count. The associated tables present the GO term ID and function for the enriched term.

Six cDNA libraries were constructed from fresh leaves of *A. spinulosa* (S) and *A. metteniana* (H), yielding a total of 231.57 Mb of clean data, with 38.60 Mb obtained for each sample (Q30 ≥ 92.6%). The mRNA abundance of each gene in each sample was profiled using FPKM method. A total of 29,060 genes exhibited an FPKM expression value above 1.0 in at least one sample (Supplementary Figure S1). The hierarchical cluster dendrogram showed the expression pattern of biological repeats be clustered together (Supplementary Figure S2). Furthermore, a total of 14,639 DEGs were identified in the H vs. S comparison, with 7,764 upregulated genes and 6,875 downregulated genes (Figure 2A). Among the five most differentially upregulated genes, three genes had defined functions: indole-3-pyruvate monooxygenase YUCCA1 (*YUCCA1*), pleiotropic drug resistance protein 1 (*PDR1*), and cholesterol 22-hydroxylase CYP90B27 (*CYP90B27*). Correspondingly, all five of the most differentially downregulated genes had defined functions: 14 kDa zinc-binding protein (*ZBP14*), expansin-A10 (*EXPA10*), protein MEN-8 (*MEN-8*), methyl jasmonate esterase 1 (*MJE1*), and mannose-specific lectin (*dfa*). The results of DEGs KEGG enrichment analysis indicated that 38 pathways were enriched (Figure 2B; Supplementary Table S3). Furthermore, GO enrichment analysis revealed that the top 20 most significant GO categories as follows: “regulation of transcription, DNA-templated”, “protein phosphorylation”, “carbohydrate metabolic process”, “defense response”, “transmembrane transport”, “membrane”, “transmembrane transporter activity”, “copper ion binding”, “iron ion binding”, “ADP binding”, “sequence-specific DNA binding”, “ATPase activity”, “monooxygenase activity”, “protein kinase activity”, “microtubule binding”, “carbohydrate binding”, “protein serine/threonine kinase activity”, “DNA binding”, “DNA-binding transcription factor activity”, “oxidoreductase activity, acting on paired donors, with incorporation or reduction of molecular” (Figure 2C).

### Identification of R genes

**FIGURE 3:**
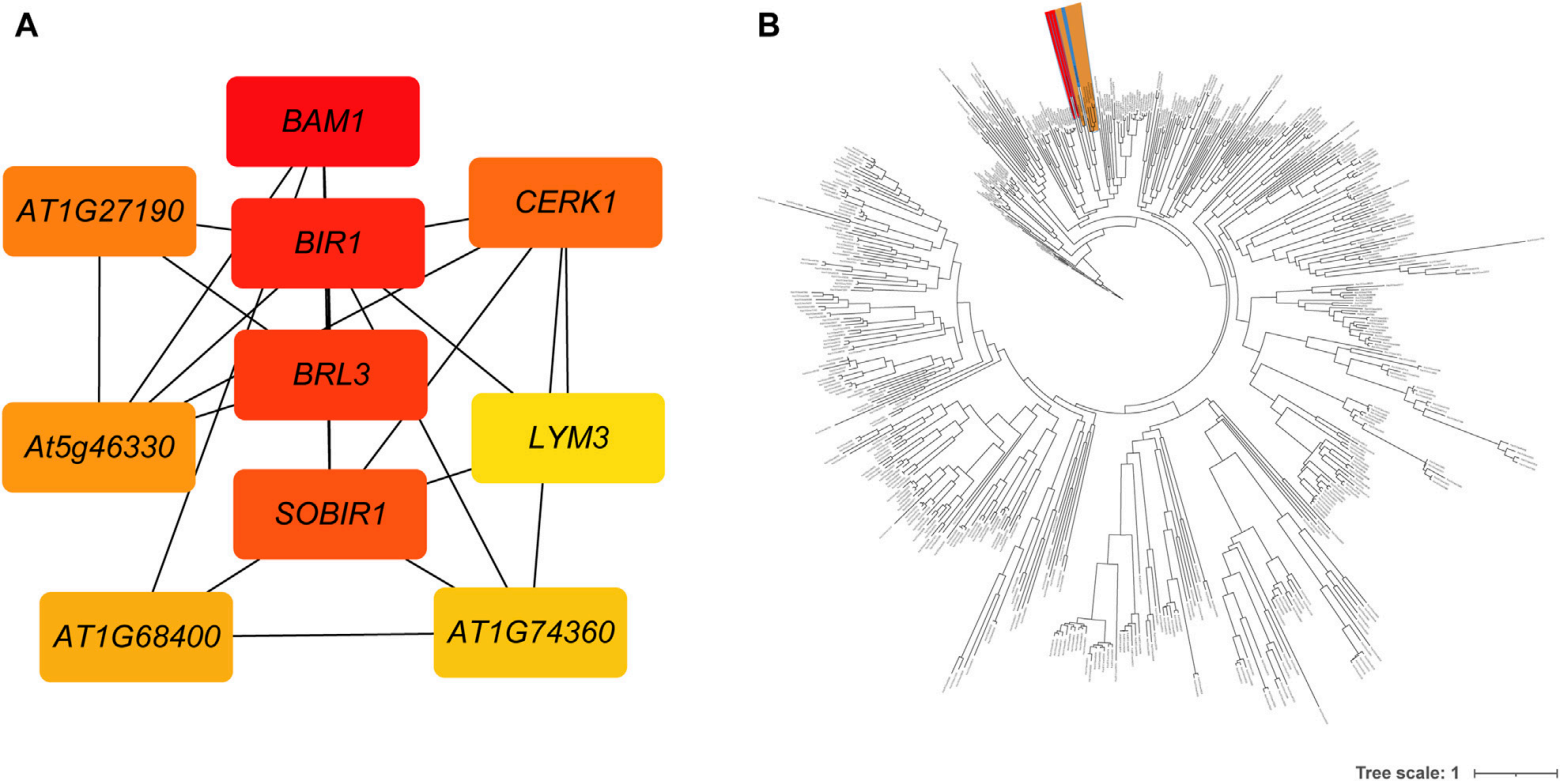
The interactions and evolutionary relationships of differentially expressed R genes. **(A)** The interaction network of top 10 nodes ranked by degree algorithm. The more forward ranking is represented by a redder color. **(B)** The maximum-likelihood phylogenetic tree of 551 RGAs. The *BAM* is highlighted in red, the *FLS2* in yellow, and the *TMK* in blue.

A total of 1,290 R genes were identified in the *A. spinulosa* genome using a genome-wide scanning pipeline (Li et al., 2016) (Supplementary Table S4). Among these R genes, 62.2% (803) belonged to the RLK category, while there were 110 NBS-related R genes, with 25 being of the TIR type. Further analysis revealed that 839 R genes were expressed in leaf tissue (Supplementary Figure S3). Of these, 606 R genes overlapped with DEGs, including 426 RLK-encoding R genes . Ten of the most differentially expressed genes were as follows: disease resistance protein L6 (*L6*), receptor-like protein kinase At1g49730 (*At1g49730*), disease resistance protein RUN1 (*RUN1*), leucine-rich repeat receptor-like serine/threonine-protein kinase BAM1 (*BAM1*), leucine-rich repeat receptor-like protein kinase At5g63930 (*At5g63930*), salt tolerance receptor-like cytoplasmic kinase 1 (*OsI_16820*), G-type lectin S-receptor-like serine/threonine-protein kinase SD2-5 (*SD25*), wall-associated receptor kinase 2 (*WAK2*), disease resistance protein RPS2 (*RPS2*) and L-type lectin-domain containing receptor kinase VIII.1 (*LECRK81*). Notably, of the differentially expressed 606 R genes, 551 genes could be further supported by known resistance genes reference from the latest PRGdb (Osuna-Cruz et al., 2017). These genes were significantly enriched in “Plant-pathogen interaction” and “MAPK signaling pathway - plant” pathway. Based on the GO enrichment analysis, these genes were assigned to 16 GO terms, such as “protein kinase activity,” “ATP binding,” “protein phosphorylation,” “defense response,” “protein binding,” and more (Supplementary Table S5). Using these 551 genes’ protein sequences as queries, 250 interactions were identified by using the STRING database (Supplementary Table S6). The first four key genes calculated by sytoHubba with the MCC algorithm were *BAM1*, inactive leucine-rich repeat receptor-like protein kinase At5g48380 (*BIR1*), receptor-like protein kinase BRI1-like 3 (*BRL3*), and leucine-rich repeat receptor-like serine/threonine/tyrosine-protein kinase SOBIR1 (*SOBIR1*) (Figure 3A). To investigate the phylogenetic relationship between R genes, we constructed an evolutionary tree. The maximum-likelihood phylogenetic tree showed that the closest relative genes of *BAM1* were LRR receptor-like serine/threonine-protein kinase FLS2 (*FLS2*) and receptor-like kinase TMK (*TMK*) (Figure 3B). PPI network also revealed that *BAM1* had interaction with *FLS2* and *TMK* (Supplementary Table S6).

### Correlation between DEGs and DAMs associated with R genes

**FIGURE 4:**
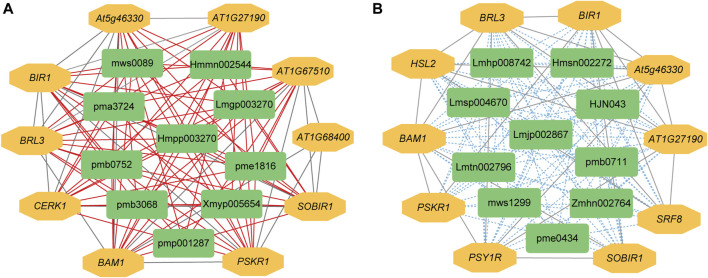
The interaction network between DEGs and DAMs associated with R genes. **(A)** Positive regulatory network. **(B)** Negative regulatory network. A red line indicates a positive correlation, whereas a blue line indicates a negative correlation between compound content and gene expression. pmp001287: N-Benzylmethylene, pmb3068: 1-*O*-p-Coumaroylquinic, pme1816: Neochlorogenic, Hmmn002544: Ferulic, Lmgp003270: Scopoletin-7-*O*-glucuronide, pma3724: 1-*O*-Feruloylquinic, pmb0752: 3-*O*-Feruloylquinic, Hmpp003270: Luteolin-4′-*O*-glucoside, mws0089: Kaempferol-7-*O*-glucoside, Xmyp005654: Kaempferol-4′-O-glucoside, Hmsn002272: Demethyl, mws1299: Luteolin-8-C-glucoside, Lmtn002796: Aromadendrin-7-*O*-glucoside, Zmhn002764: 4-*O*-(6′-*O*-Glucosylcaffeoyl)-3,4-dihydroxybenzoic, Lmhp008742: LysoPC, pme0434: Procyanidin, Lmsp004670: Kaempferol-3-*O*-glucoside-7-*O*-rhamnoside, Lmjp002867: Kaempferol-3-*O*-neohesperidoside, pmb0711: Quercetin-7-*O*-rutinoside, HJN043: Cinnamtannin.

The expression levels of 14,097 DEGs were significantly correlated with the abundance of 111 DAMs. Further analysis showed that 585 significantly differentially expressed R genes were significantly positively correlated with 111 DAMs, while 574 significantly differentially expressed R genes were significantly negatively correlated with 111 DAMs (Supplementary Tables S7, S8). Subsequently, we constructed interaction networks between R genes and DAMs exhibiting significantly positive correlations, as well as for R genes and DAMs showing significantly negative correlations, respectively. According to the results of cytoHubba, in the significantly positively correlated network, the top 10 core genes are probably inactive leucine-rich repeat receptor-like protein kinase At5g48380 (*BIR1*), receptor-like protein kinase BRI1-like 3 (*BRL3*), *BAM1*, phytosulfokine receptor 1 (*PSKR1*), leucine-rich repeat protein kinase family protein (*AT1G27190*), leucine-rich receptor-like protein kinase family protein (*At5g46330*), SOBIR1, lysm-containing receptor-like kinase 1 (*LYK1*, also named as *CERK1*), leucine-rich repeat transmembrane protein kinase family protein (*AT1G68400*), and Leucine-rich repeat protein kinase family protein (*AT1G67510*). The first 10 core metabolites are N-Benzylmethylene isomethylamine, 1-*O*-p-Coumaroylquinic acid, Neochlorogenic acid (5-*O*-Caffeoylquinic acid), Ferulic acid-4-*O*-glucoside, Scopoletin-7-*O*-glucuronide, 1-*O*-Feruloylquinic acid, 3-*O*-Feruloylquinic acid, Luteolin-4′-*O*-glucoside, Kaempferol-7-*O*-glucoside, and Kaempferol-4′-*O*-glucoside. In the significantly negatively correlated network, the top 10 core genes are *BIR1*, *BRL3*, *BAM1*, *At5g46330*, *AT1G27190*, *SOBIR1*, Leucine-rich receptor-like protein kinase family protein (*PSY1R*), STRUBBELIG-receptor family 8 (*SRF8*), LRR receptor-like serine/threonine-protein kinase HSL2 (*HSL2*), and *PSKR1*. The first 10 core metabolites are Demethyl coniferin, Luteolin-8-C-glucoside (Orientin), Aromadendrin-7-*O*-glucoside, 4-O-(6′-*O*-Glucosylcaffeoyl)-3,4-dihydroxybenzoic acid, LysoPC 20:5, Procyanidin B2, Kaempferol-3-*O*-glucoside-7-*O*-rhamnoside, Kaempferol-3-*O*-neohesperidoside, Quercetin-7-*O*-rutinoside, and Cinnamtannin B1 (Figures 4A, B).

### Verification of RNA-Seq gene expression

We utilized qRT-PCR to validate the gene expression profiles identified through Illumina sequencing analysis. Ten DEGs were randomly selected for validation, including wall-associated receptor kinase-like 1, leucine-rich repeat receptor-like serine/threonine-protein kinase BAM2, *FLS2*, and others (Supplementary Tables S9, S10). The gene expression of all 10 DEGs in the qRT-PCR experiment was consistent with the expression patterns in RNA-Seq results, with a Pearson correlation coefficient of the fold change between the qRT-PCR experiment and RNA-Seq being 0.78 (Supplementary Figure S4). These results confirmed the reliability of Illumina sequencing in this experiment.

## Discussion

Tree ferns serve as a crucial evolutionary link between lower and higher plant species (Cao et al., 2017). They are widely utilized globally for ornamental, medicinal, and occasionally culinary purposes, but face challenges such as overexploitation, climate change, harsh living conditions, and insect pests (Dadang et al., 2020). These threats have led to 94 Alsophila species being listed on the IUCN Red List, emphasizing the urgent need for conservation efforts. R genes, key players in plant defense mechanisms, typically encode proteins with conserved domains like nucleotide-binding site, leucine-rich repeat, and Toll/interleukin-1 receptor (van Ooijen et al., 2007). These genes provide resistance against a diverse array of organisms including bacteria, viruses, fungi, oomycetes, nematodes, and insects (Kaloshian, 2004). R gens have been investigated in multiple important plants, such sorghum (Zhang et al., 2022a), soybean (Wang et al., 2021b), and rice (Wang et al., 2019). In sorghum, a total of 308 R genes have been identified, among which three R genes, including G325100 (NBS-LRR), G131600 (RLK), and G181300 (RLK), were confirmed to be upregulated in response to aphids using quantitative real-time PCR (Zhang et al., 2022b). Furthermore, in a study of a highly resistant selection eggplant genome, 1023 R genes were identified, with 15 R genes overlapping with positively selected genes, likely playing a key role in eggplant self-defense (Li et al., 2021). Enhanced comprehension of R genes can facilitate the development of more effective strategies to safeguard the survival of tree ferns in the face of environmental challenges. Pest-resistant rice could be developed through the crossbreeding of plants expressing the *Xa21* gene with those expressing both a Bt gene (Pandolfi et al., 2017). We are looking forward to developing insect-resistant *Alsophila* to safeguard this rare plant.

In this study, we present the metabolomics and transcriptomic analysis of leaf tissues from two representative tree fern species, *A. spinulosa* and *A. metteniana*, focusing on the exploration of R genes. Our metabolomics analysis revealed a high abundance of the metabolite skimmin in both *A. spinulosa* and *A. metteniana*. Skimmin is known to exhibit numerous bioactive and pharmacological properties, which may be closely linked to the medicinal significance of Alsophila species (Zhang et al., 2020; Sun et al., 2023). The DAMs were significantly enriched in various pathways, including 2-oxocarboxylic acid metabolism. Interestingly, 2-oxocarboxylic acid metabolism has been highlighted as a top-5 KEGG enriched pathway in drought-stressed sugarcane experiment (Yang et al., 2022) and has been reported to be associated with cell death and immunity in rice (Zhang et al., 2022a). The potential connection of 2-oxocarboxylic acid metabolism to the distinct ecological niches of *A. spinulosa* and *A. metteniana* is worth exploring further. Accordingly, a total of 14,639 DEGs were obtained. The GO enrichment analysis of DEGs revealed that defense response was one of the top 20 most significant GO categories. In the *A. spinulosa* genome, a total of 1,290 R genes were identified, with 606 of them being DEGs. Both the KEGG and GO enrichment analyses indicated that these genes are significantly associated with disease resistance, including pathways like plant-pathogen interaction and GO terms related to defense response. Gene networks play a crucial role in understanding biological processes and reactions in organisms. The interactions network analysis revealed that *BAM1* was one of the first four key genes. *BAM1* has been reported to be involved in regulation of leaf shape, size and symmetry in Arabidopsis (DeYoung et al., 2006). On the other hand, *BAM1* was also involved in virus-host interactions in Tobacco (Tran and Citovsky, 2021). A precise gene phylogenetic tree is essential for inferring the origin of genes, detecting molecular adaptation, and understanding the evolution of morphological characters (Kapli et al., 2020). The maximum-likelihood phylogenetic tree indicates a close relationship between *BAM1*, *FLS2*, and *TMK*, as supported by PPI network analysis results. *FLS2* has been known to enhance disease resistance in crop plants while *TMK* plays a role in orchestrating plant growth in Arabidopsis (Dai et al., 2013; Wei et al., 2020). The study of interactions between cellular macromolecules is fundamental to the understanding of biological systems (Raman, 2010). In the gene-metabolite interaction networks *BAM1* emerges as the core gene, underscoring its significance in the *Alsophila* species biological system. The correlation analysis indicates that *BAM1* is positively correlated with DAMs of neochlorogenic acid and kaempferol-7-*O*-glucoside. Neochlorogenic acid exhibits antioxidant, antifungal, anti-inflammatory, and anticarcinogenic effects (Navarro-Orcajada et al., 2021), while Kaempferol-7-*O*-glucoside has been reported to possess antioxidant and anti-inflammatory properties (Wang et al., 2018). Taken together, *BAM1* may indeed have a significant impact on the biology of *Alsophila* species.

This is the first study to characterize R genes in *Alsophila* species. The construction of a pan-genome can serve as a powerful tool for exploring genomic evolution, the emergence and domestication of species, and providing valuable insights for enhancing plant traits (Li et al., 2022b). A pan-genome study on sorghum revealed high levels of diversity among five sorghum accessions in R genes (Wang et al., 2021a). Additionally, the pan-NLRome reported in *Arabidopsis* indicated that a high diversity of NLR-integrated domains favor known virulence targets (Van de Weyer et al., 2019). This suggests that attention should be given not only to conserved regions but also to genetic variations among different breeds. Therefore, exploring the pan-genome in Alsophila species may offer a promising future direction for studying the functions of R genes. The comprehensive analysis of global metabolic and transcriptomic changes in the leaves of *A. spinulosa* and *A. metteniana* broaden our understanding of resistance genes in tree ferns.

## Data Availability

The datasets presented in this study can be found in online repositories. The names of the repository/repositories and accession number(s) can be found in the article/Supplementary Material.
