# Supplementary material for: Transcriptomics and metabolomics analyses provide insights into resistance genes of tree ferns
Source: Front Genet. 2024 Jun 10;15:1398534. doi: 10.3389/fgene.2024.1398534 (PMC11194355; doi:10.3389/fgene.2024.1398534)
Supplement: Supplementary file 2 [file Table1.DOCX]

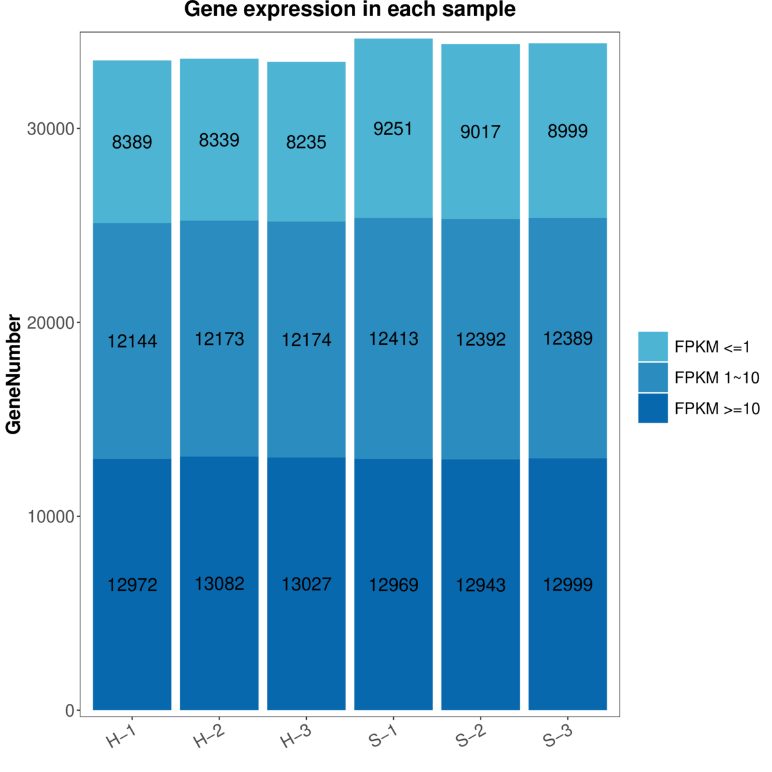


**Figure S1. Statistic of expressed genes.**


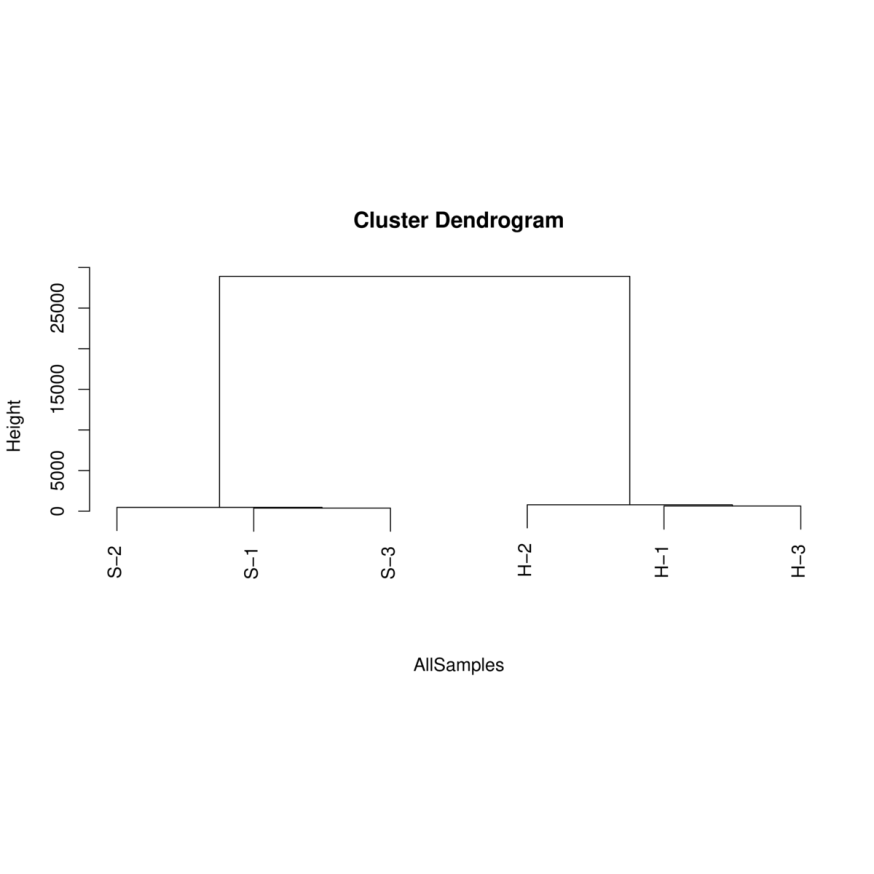


**Figure S2. Dendrogram of cluster analysis by method of Ward linkage for 6 samples.**

**
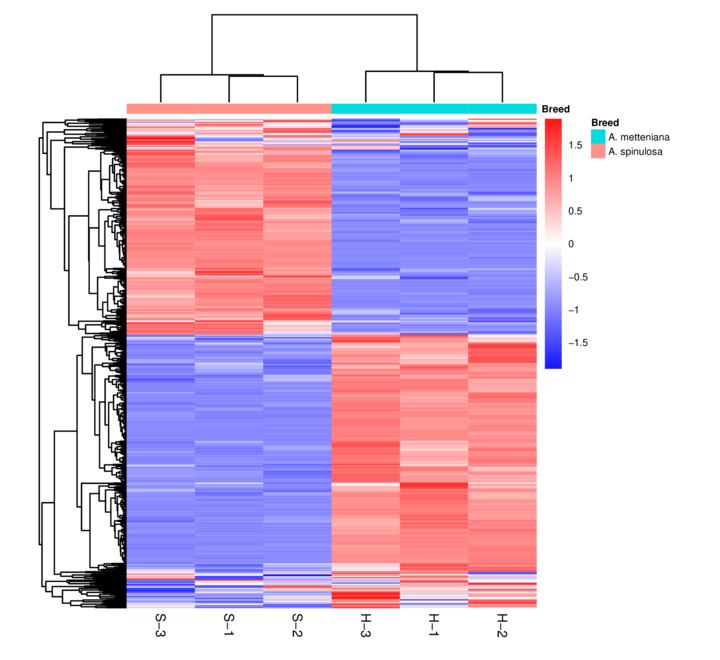
**

**Figure S3. Cluster heatmap of RGAs.** Each column represents a sample, and each row represents an RGA.

**
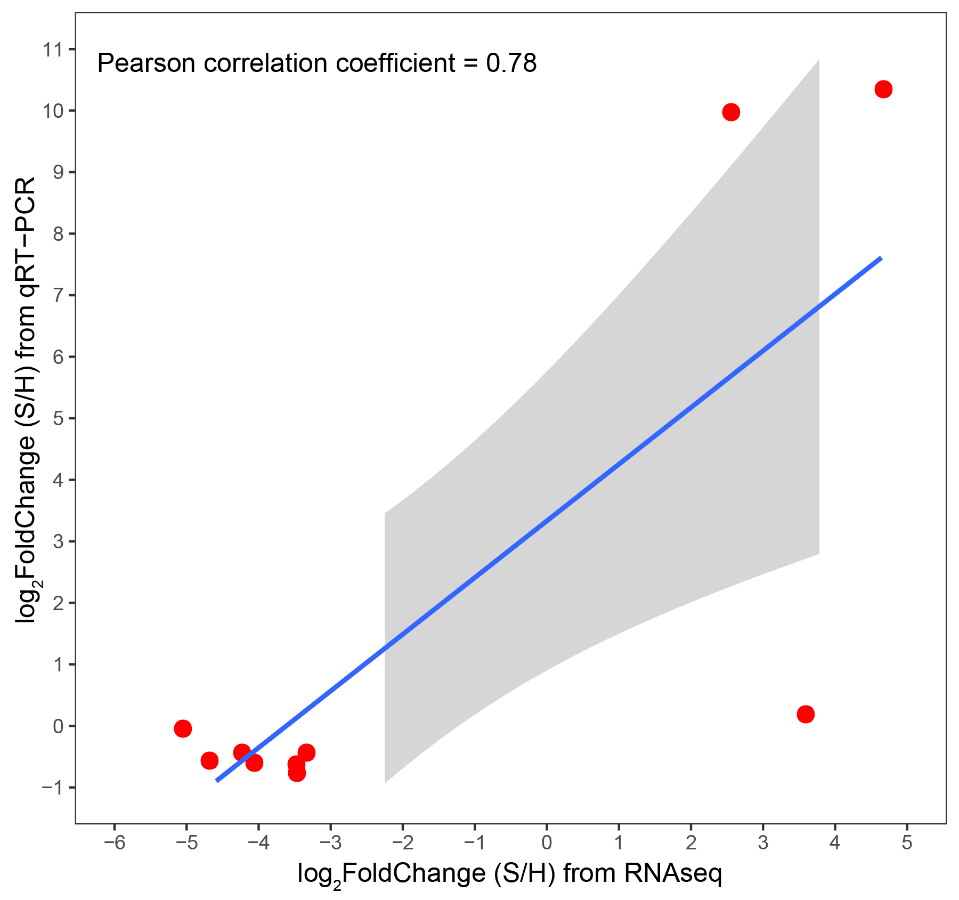
**

**Figure S4. Correlation of gene expression results obtained from qRT-PCR analysis and RNA-Seq.**The blue curve represents the fitting curve. A red dot represents a gene.
